# Avelumab in patients with previously treated metastatic adrenocortical carcinoma: phase 1b results from the JAVELIN solid tumor trial

**DOI:** 10.1186/s40425-018-0424-9

**Published:** 2018-10-22

**Authors:** Christophe Le Tourneau, Christopher Hoimes, Corrine Zarwan, Deborah J. Wong, Sebastian Bauer, Rainer Claus, Martin Wermke, Subramanian Hariharan, Anja von Heydebreck, Vijay Kasturi, Vikram Chand, James L. Gulley

**Affiliations:** 1 Department of Medical Oncology, Institut Curie, 26, rue d’ulm, 75005 Paris & Saint-Cloud, France; 2 Versailles Saint Quentin en Yvenlines University, Montigny-le-Bretonneux, France; 3 INSERM U900 Research Unit, Saint-Cloud, France; 4 Case Western Reserve University and University Hospitals Seidman Cancer Center, Cleveland, OH USA; 5 Lahey Hospital and Medical Center, Burlington, MA USA; 6 UCLA Department of Medicine, California, Los Angeles USA; 7 Department of Medical Oncology, West German Cancer Centre, University of Duisburg-Essen, Hufelandstraße, Essen, Germany; 8 German Cancer Consortium, Partner Site University Hospital Essen, Essen, Germany; 9 Department of Hematology, Oncology and Stem Cell Transplantation, University Medical Center, Faculty of Medicine, University of Freiburg, Freiburg, Germany; 10 Present address: Department of Hematology and Oncology, Augsburg Medical Center, Augsburg, Germany; 11 Early Clinical Trial Unit, University Cancer Center, Dresden, Germany; 12 Pfizer, Inc, New York, NY USA; 13 Merck KGaA, Darmstadt, Germany; 14 EMD Serono Inc, Rockland, MA USA; 15 EMD Serono Research and Development Institute, Billerica, MA USA; 16 National Cancer Institute, National Institutes of Health, Bethesda, MD USA

**Keywords:** Avelumab, Adrenocortical carcinoma, PD-L1, Phase 1, JAVELIN solid tumor, Neuroendocrine tumors

## Abstract

**Background:**

We assessed the efficacy and safety of avelumab, an anti-programmed death ligand 1 (PD-L1) antibody, in patients with previously treated metastatic adrenocortical carcinoma (mACC).

**Methods:**

In this phase 1b expansion cohort, patients with mACC and prior platinum-based therapy received avelumab at 10 mg/kg intravenously every 2 weeks. Continuation of mitotane was permitted; however, mitotane levels during the study were not recorded. Tumor response was assessed by Response Evaluation Criteria In Solid Tumors v1.1.

**Results:**

Fifty patients received avelumab and were followed for a median of 16.5 months. Prior treatment included ≥2 lines in 74.0%; mitotane was continued in 50.0%. The objective response rate (ORR) was 6.0% (95% CI, 1.3% to 16.5%; partial response in 3 patients). Twenty-one patients (42.0%) had stable disease as best response (disease control rate, 48.0%). Median progression-free survival was 2.6 months (95% CI, 1.4 to 4.0), median overall survival (OS) was 10.6 months (95% CI, 7.4 to 15.0), and the 1-year OS rate was 43.4% (95% CI, 27.9% to 57.9%). In evaluable patients with PD-L1+ (*n* = 12) or PD-L1− (*n* = 30) tumors (≥5% tumor cell cutoff), ORR was 16.7% vs 3.3% (*P* = .192). Treatment-related adverse events (TRAEs) occurred in 82.0%; the most common were nausea (20.0%), fatigue (18.0%), hypothyroidism (14.0%), and pyrexia (14.0%). Grade 3 TRAEs occurred in 16.0%; no grade 4 to 5 TRAEs occurred. Twelve patients (24.0%) had an immune-related TRAE of any grade, which were grade 3 in 2 patients (4.0%): adrenal insufficiency (*n* = 1), and pneumonitis (*n* = 1).

**Conclusions:**

Avelumab showed clinical activity and a manageable safety profile in patients with platinum-treated mACC.

**Trial registration:**

Clinicaltrials.gov NCT01772004; registered January 21, 2013.

**Electronic supplementary material:**

The online version of this article (10.1186/s40425-018-0424-9) contains supplementary material, which is available to authorized users.

## Background

Adrenocortical carcinoma (ACC) is a rare endocrine tumor with an incidence of approximately 0.7 cases/1 million population per year [1]. Genomic studies indicate that mutations in defined driver genes, including zinc and ring finger 3 (*ZNRF3*)*,* telomerase reverse transcriptase (*TERT*)*,* protein kinase cAMP-dependent type I regulatory subunit alpha (*PRKAR1A*), ribosomal protein L22 (*RPL22*), telomeric repeat binding factor 2 (*TERF2*), cyclin E1 (*CCNE1*), and neurofibromin 1 (*NF1*), may have a major role in the etiology of ACC [2], and that ACC has a moderate overall mutation burden compared with other tumor types [3]. Patients with ACC have a poor prognosis, with an estimated 5-year survival rate for metastatic ACC (mACC) of < 20% [4–6]. Therapeutic options for mACC are limited and include surgery; platinum agents, mitotane, streptozocin, and other systemic therapies; and locoregional radiotherapy [7]. Although cytotoxic treatments are widely administered, they are associated with limited efficacy and high toxicity, and targeted agents have not shown clinically meaningful activity in this disease [8]. Thus, there is an urgent need for new treatment options.

Cancer cells can exploit the programmed death 1 (PD-1)/programmed death ligand 1 (PD-L1) immune checkpoint pathway to promote an immunosuppressive environment and allow tumor growth. Specifically, PD-L1 expressed on tumor cells binds to PD-1 expressed on activated T cells, resulting in downregulation of antitumor immune responses [9, 10]. Monoclonal antibodies targeting the PD-1/PD-L1 axis have shown clinical activity in multiple tumor types [11]. PD-L1 is expressed in ACC tissues throughout various stages of disease, providing a rationale for assessing anti–PD-1/PD-L1 antibodies in mACC [12].

Avelumab is a human IgG1 monoclonal antibody with a wild-type Fc region that was designed to specifically bind and block PD-L1 [13]. Unlike other anti–PD-L1/PD-1 antibodies in clinical practice, avelumab is unique in that preclinical models show that it can induce antibody-dependent cellular cytotoxicity of tumor cells [14, 15]. Thus, avelumab may engage both the adaptive and innate immune systems. Avelumab treatment has been associated with durable responses in several tumor types [16–18], and avelumab has been approved in various countries for the treatment of patients with metastatic Merkel cell carcinoma and in the United States and Canada for treatment of locally advanced or metastatic urothelial carcinoma that progressed during or after platinum-containing chemotherapy [19]. Here we report results from a phase 1b cohort of patients with previously treated mACC who received avelumab.

## Methods

### Study design and treatment

JAVELIN Solid Tumor (ClinicalTrials.gov ID NCT01772004) in an international, multicenter, phase 1, open-label trial designed to investigate the clinical activity, safety, and pharmacokinetics of avelumab in patients with metastatic solid tumors, with expansion in selected tumor types. Findings from the dose-escalation portion of this study (phase 1a) were reported previously [13]. In this phase 1b dose-expansion cohort, eligible patients were aged ≥18 years and had an Eastern Cooperative Oncology Group performance status (ECOG PS) of ≤1; histologically or cytologically confirmed mACC with ≥1 measurable lesion; ≥1 line of prior systemic therapy for metastatic disease (including ≥1 platinum-based therapy); and relapsed, refractory, or progressive disease following last line of treatment. Patients receiving mitotane were permitted to continue treatment during the trial; however, details relating to ongoing mitotane treatment was not systematically collected and mitotane levels during the study were not recorded. Patients were also required to have adequate hematologic, hepatic, and renal function and availability of a fresh or archival tumor specimen. Exclusion criteria included prior treatment with a T cell-targeting immune checkpoint inhibitor, other cancer diagnosis within 5 years, rapidly progressive disease, CNS metastases, known autoimmune disease, or ongoing steroid treatment (except for patients with adrenal insufficiency, who could continue treatment at a physiological replacement dose).

The trial was conducted in accordance with the ethics principles of the Declaration of Helsinki and the International Council for Harmonisation Guidelines on Good Clinical Practice. The protocol was approved by the institutional review board or independent ethics committee of each center. All patients provided written informed consent before enrollment.

### Procedures

Avelumab (EMD Serono, Rockland, MA) 10 mg/kg was administered intravenously over 60 mins every 2 weeks until progression, unacceptable toxicity, or other protocol-based criteria for withdrawal occurred. Dose modifications were not permitted. Treatment was discontinued following: 1) any grade 4 adverse event (AE) except for single laboratory values out of the normal range that were unrelated to study treatment, without clinical correlate, and resolved within 7 days with medical management; 2) any grade 3 AE except for: (i) transient (≤6 h) influenza-like symptoms or pyrexia controlled with medical management; (ii) fatigue, local infusion-related reaction, headache, nausea, or emesis that resolved to grade ≤ 1 within 24 h; (iii) single laboratory values out of the normal range that were unrelated to study treatment and without clinical correlate (excluding grade ≥ 3 increase in liver enzyme concentrations) that resolved to grade ≤ 1 within 7 days; (iv) tumor flare phenomena (local pain, irritation, or localized rash at a tumor site); (v) grade ≥ 3 amylase or lipase abnormality not associated with symptoms or clinical manifestations of pancreatitis; or 3) increase in Eastern Cooperative Oncology Group performance status to ≥3 not resolved to ≤2 by Day 14 of the following cycle. Grade 2 AEs were managed by dose delays; events that did not resolve to grade ≤ 1 by the end of the next treatment cycle or recurred led to permanent discontinuation of avelumab (except for hormone insufficiencies that could be managed by replacement therapy). A premedication regimen of diphenhydramine and acetaminophen was administered 30 to 60 min before all avelumab infusions.

Safety was assessed at each biweekly trial visit and included assessment of AEs, physical examination, and clinical laboratory tests. AEs and laboratory abnormalities were graded according to the National Cancer Institute Common Terminology Criteria for Adverse Events v4.0. A serious AE (SAE) was defined as any untoward event that was life-threatening, required hospitalization, resulted in disability, was a congenital anomaly, or resulted in death. Immune-related AEs were identified using a prespecified list of Medical Dictionary for Regulatory Activities terms.

Clinical activity was assessed by investigators using Response Evaluation Criteria In Solid Tumors (RECIST) v1.1 [20] and modified immune-related response criteria [21]. Radiographic tumor assessments were completed at baseline and then every 6 weeks. For patients who had a partial response (PR) or complete response (CR), a confirmatory computed tomography or magnetic resonance imaging scan was completed ≥28 days later, preferably at the scheduled 6-week interval. PD-L1 expression was assessed using a proprietary immunohistochemistry assay (Dako PD-L1 IHC 73–10; Dako, Carpinteria, CA) [18].

### Outcomes

Primary endpoints for the whole JAVELIN Solid Tumor trial are the occurrence of dose-limiting toxicities during the first 3 weeks of treatment in the phase 1a dose-escalation part (reported previously) [13] and confirmed best overall response adjudicated by an independent review committee in specified expansion cohorts. Prespecified endpoints assessed in this cohort included best overall response per investigator assessment (defined as best response per RECIST v1.1 obtained among all tumor assessments after the start of treatment with avelumab until documented disease progression), immune-related best overall response, duration of response (defined as the time from first documented PR or CR until progressive disease or death, whichever occurred first), progression-free survival (PFS), overall survival (OS), evaluation of PD-L1 expression, and safety (including incidence and severity of AEs). Change in the sum of target lesion diameters from baseline over time was evaluated in patients with baseline tumor assessments and ≥ 1 postbaseline assessment.

### Statistical analysis

Enrollment of 50 patients was planned for this cohort; this sample size was selected to provide point estimates and 95% Clopper-Pearson CIs for an objective response rate (ORR; proportion of patients with a PR or CR) of 10% (95% CI, 3.3% to 21.8%) in the case of 5 responders, and 20% (95% CI, 10.0% to 33.7%) in the case of 10 responders. Safety and clinical activity were analyzed in all patients who received ≥1 dose of avelumab. Time-to-event endpoints were estimated with the Kaplan-Meier method, and CIs for the median were calculated using the Brookmeyer-Crowley method. *P* values for association between categorical variables were determined using the Fisher exact test.

## Results

Between September 9, 2014, and the data cutoff date of December 31, 2016, 50 patients were enrolled in 6 countries. Median age was 50 years (range, 21 to 71 years) (Table 1). Patients were heavily pretreated; the median number of prior lines of systemic therapy was 2 (range, 1 to 6), and 37 patients (74.0%) had received ≥2 prior lines. Date of disease progression before enrollment was documented in 49 patients, and median time since last disease progression was 0.92 months (range, 0.33 to 8.61 months). Twenty-five patients (50%) continued to receive concurrent mitotane. Median duration of avelumab treatment was 3.4 months (range, 0.5 to 24.8 months), and median follow-up was 16.5 months (range, 11.7 to 27.6 months). Patients received a median 7 doses of avelumab (range, 1 to 52). At data cutoff, 5 patients (10.0%) remained on treatment. Reasons for treatment discontinuation were disease progression (*n* = 32 [64.0%]), AE (*n* = 5 [10.0%]), death (*n* = 3 [6.0%]), withdrawal of consent (*n* = 2 [4.0%]), protocol noncompliance (*n* = 1 [2.0%]), and other (*n* = 2 [4.0%]).

**Table 1:** Patient Demographics and Baseline Characteristics

Characteristics	(*N* = 50)
Age, median (range), years	50.0 (21–71)
< 65 years, n (%)	46 (92.0)
≥ 65 years, n (%)	4 (8.0)
Sex, n (%)	
Male	24 (48.0)
Female	26 (52.0)
Geographic region, n (%)	
USA	27 (54.0)
Europe	22 (44.0)
Asia	1 (2.0)
ECOG PS, n (%)	
0	19 (38.0)
1	31 (62.0)
Median time since first diagnosis (range), years	1.6 (0.2–15.1)
Median time since last disease progression (range), months	0.92 (0.33–8.61)
Number of prior lines of systemic anticancer therapy, n (%)	
1	13 (26.0)
2	18 (36.0)
3	10 (20.0)
≥ 4	9 (18.0)
Receiving concomitant mitotane, n (%)	25 (50.0)
PD-L1 expression on tumor cells (≥1% cutoff), n (%)	
Negative	27 (54.0)
Positive	15 (30.0)
Not evaluable	8 (16.0)
PD-L1 expression on tumor cells (≥5% cutoff), n (%)	
Negative	30 (60.0)
Positive	12 (24.0)
Not evaluable	8 (16.0)

**Fig. 1:**
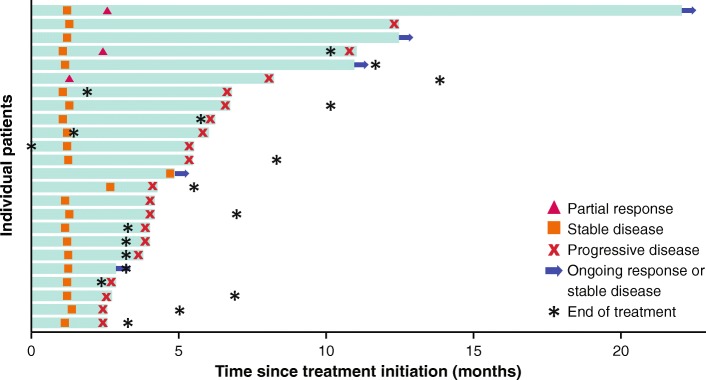
Time to and duration of response or stable disease (*n* = 24). Bars represent individual patients with partial response or stable disease. At time of data cutoff, partial response was ongoing in 1 patient

**Fig. 2:**
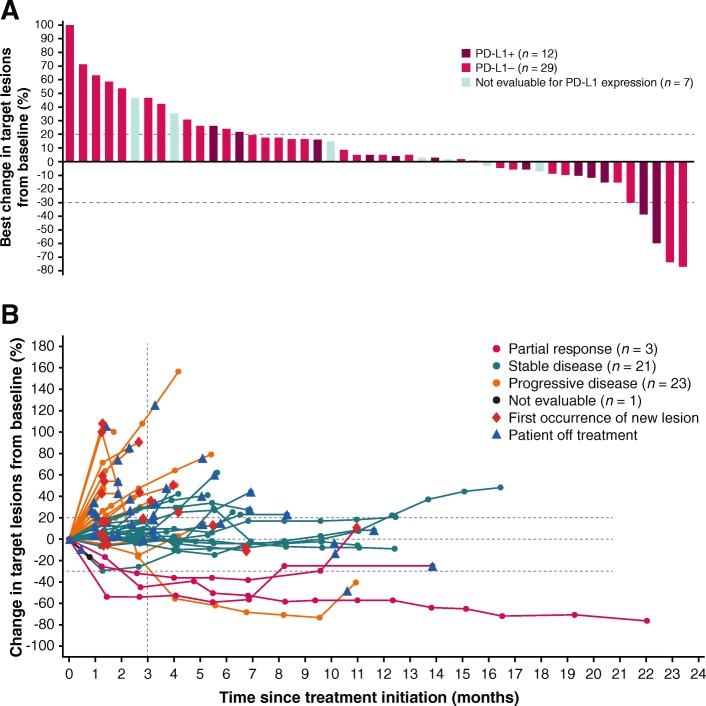
**a** Best change and **b** change over time from baseline in sum of target lesion diameters in evaluable patients (*n* = 48). In **a** bars for individual patients are color-coded according to tumor PD-L1 status based on a 5% PD-L1 expression cutoff in tumor cells. In **b** lines for individual patients are color-coded based on confirmed best overall response

Of 50 patients enrolled, 3 had confirmed PRs, and the ORR was 6.0% (95% CI, 1.3% to 16.5%) (Table 2). Of the 3 responding patients, 2 were receiving concomitant mitotane (both had prior progression while receiving mitotane), and 2 had PD-L1+ tumors (based on 1% or 5% expression cutoffs). In patients who had received 1 (*n* = 13), 2 (*n* = 18), or ≥ 3 (*n* = 19) prior lines of systemic therapy since diagnosis, ORR was 15.4%, 5.6%, and 0%, respectively. Response was ongoing in 1 patient, who had a duration of response of 19.4 months at data cutoff (Fig. 1); this was a man aged 63 years with a PD-L1− tumor who had received prior treatment with cisplatin, etoposide, and mitotane and had target lesions in the liver and lymph node (Additional file 1: Figure S1). The patient received avelumab with concurrent mitotane, had a PR documented at the second assessment (week 13), and remained on treatment without progression throughout 22.0 months of follow-up. Twenty-one patients had a best response of stable disease, resulting in a disease control rate of 48.0%, which was the same in patients with or without concomitant mitotane. Using immune-related response criteria, ORR was 6.0% (95% CI, 1.3% to 16.5%), and the disease control rate was 58.0% (Additional file 1: Table S1). Of 48 patients evaluable for change in tumor size, 16 (33.3%) had a reduction of any level vs baseline, including a reduction of ≥30% in 4 patients (8.0%) (Fig. 2a). One patient had an initial assessment showing progressive disease (~ 100% increase vs baseline and new lesion at week 7) but later assessments showed a decrease vs baseline (nadir − 74%; Fig. 2b).

**Table 2:** Confirmed Best Overall Response Based on RECIST v1.1

Response	With Mitotane (*n* = 25)	Without Mitotane (*n* = 25)	Overall (*N* = 50)
Confirmed best overall response, n (%)			
Complete response	0	0	0
Partial response	2 (8.0)	1 (4.0)	3 (6.0)
Stable disease	10 (40.0)	11 (44.0)	21 (42.0)
Progressive disease	10 (40.0)	13 (52.0)	23 (46.0)
Not evaluable	3 (6.0)	0	3 (6.0)
ORR (95% CI), %	8.0 (1.0–26.0)	4.0 (0.1–20.4)	6.0 (1.3–16.5)
Disease control rate, %	48.0	48.0	48.0

**Fig. 3:**
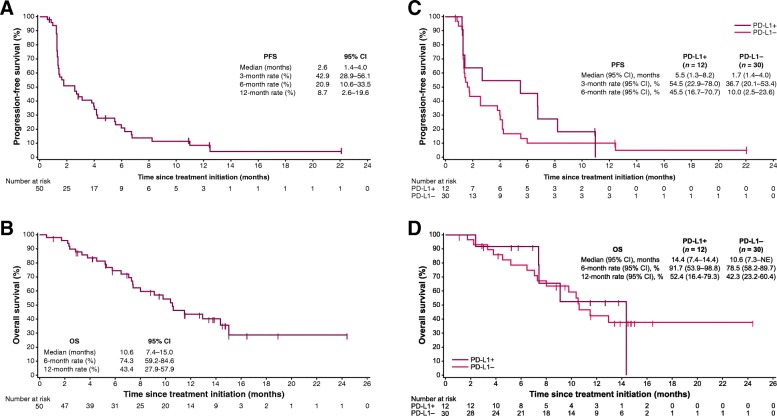
**a** PFS and **b** OS in the overall population (*N* = 50), and **c** PFS and **d** OS based on PD-L1 expression on tumor cells (5% cutoff) in evaluable patients (*n* = 42). NE, not evaluable

Median PFS was 2.6 months (95% CI, 1.4 to 4.0), and PFS rates at 6 months and 1 year were 20.9% (95% CI, 10.6% to 33.5%) and 8.7% (95% CI, 2.6% to 19.6%), respectively (Fig. 3a). Using immune-related criteria, median PFS was 3.8 months (95% CI, 2.4 to 5.5). Median OS was 10.6 months (95% CI, 7.4 to 15.0), and the 1-year OS rate was 43.4% (95% CI, 27.9% to 57.9%) (Fig. 3b).

PD-L1 expression was evaluable in tumor samples from 42 patients (84%). In patients with PD-L1+ (*n* = 12) or PD-L1– (*n* = 30) tumors based on a 5% PD-L1 expression cutoff, ORR (95% CI) was 16.7% (2.1% to 48.4%) vs 3.3% (0.1% to 17.2%) (*P* = .192), median PFS was 5.5 (1.3 to 8.2) vs 1.7 (1.4 to 4.0) months (HR, 0.66 [95% CI, 0.32 to 1.39]), and median OS was 14.4 (7.4 to 14.4) vs 10.6 (7.3 to not evaluable) months (HR, 0.91 [95% CI, 0.33 to 2.49]) (Fig. 3c and Fig. 3d). Findings were similar based on analyses of PD-L1 expression using a 1% cutoff (Additional file 1**:** Table S2 and Figure S2). Analyses of higher tumor cell PD-L1 cutoffs and PD-L1 expression on tumor-infiltrating immune cells were not informative because few patients had PD-L1+ tumors (0 to 5 patients).

All 50 patients (100.0%) had an AE of any grade; 41 patients (82.0%) had a treatment-related AE (TRAE) (Table 3). The most common TRAEs of any grade were nausea (*n* = 10; 20.0%), fatigue (*n* = 9; 18.0%), hypothyroidism (*n* = 7; 14.0%), and pyrexia (*n* = 7; 14.0%). Five patients (10%) had an infusion-related reaction; all were grade 1 or 2. Eight patients (16.0%) had a grade 3 TRAE and no patient had a grade 4 or 5 TRAE. Of patients with or without concomitant mitotane treatment during avelumab treatment (*n* = 25 in each group), 6 (24.0%) vs 2 (8.0%) patients had a grade 3 TRAE, respectively. Increase in alanine aminotransferase level (*n* = 2) was the only grade 3 TRAE that occurred in more than 1 patient; both patients received concomitant mitotane. Treatment-related grade 3 elevations in liver enzyme levels occurred only in patients who received concomitant mitotane. Twelve patients (24.0%) had an immune-related TRAE of any grade; those occurring in ≥1 patient were hypothyroidism (*n* = 7), adrenal insufficiency (*n* = 3), and pneumonitis (*n* = 2). Two patients (4.0%) had a grade 3 immune-related TRAE: adrenal insufficiency (*n* = 1) and pneumonitis (*n* = 1). Thirty-two patients (64.0%) had an SAE; in 4 patients (8.0%), the SAE was treatment related: pneumonitis (*n* = 1), adrenal insufficiency and transaminitis (*n* = 1), thyroiditis (n = 1), and pyrexia (*n* = 1). Six patients (12.0%) had AEs that led to death, but none were treatment related: disease progression (*n* = 4), failure to thrive (*n* = 1), and respiratory failure (*n* = 1).

**Table 3:** TRAEs by Concomitant Mitotane Treatment (any grade in ≥10% of patients in either group or grade 3 in any patient)

TRAE	With Mitotane (*n* = 25)		Without Mitotane (*n* = 25)	
	Any Grade	Grade 3	Any Grade	Grade 3
Any TRAE, n (%)	21 (84.0)	6 (24.0)	20 (80.0)	2 (8.0)
Nausea	7 (28.0)	0	3 (12.0)	0
Fatigue	5 (20.0)	0	4 (16.0)	0
Pyrexia	5 (20.0)	0	2 (8.0)	0
ALT increased	4 (16.0)	2 (8.0)	1 (4.0)	0
AST increased	4 (16.0)	1 (4.0)	1 (4.0)	0
Asthenia	3 (12.0)	0	1 (4.0)	0
Back pain	3 (12.0)	0	0	0
Chills	3 (12.0)	0	2 (8.0)	0
Diarrhea	3 (12.0)	0	2 (8.0)	0
Hypothyroidism	3 (12.0)	0	4 (16.0)	0
Adrenal insufficiency	2 (8.0)	1 (4.0)	1 (4.0)	0
GGT increase	2 (8.0)	1 (4.0)	0	0
Vomiting	2 (8.0)	0	4 (16.0)	0
Anemia	1 (4.0)	1 (4.0)	2 (8.0)	0
Hypophosphatemia	1 (4.0)	0	2 (8.0)	1 (4.0)
Infusion-related reaction	1 (4.0)	0	4 (16.0)	0
Lymphopenia	1 (4.0)	1 (4.0)	0	0
Transaminases increased	1 (4.0)	1 (4.0)	0	0
Pneumonitis	0	0	2 (8.0)	1 (4.0)

Abbreviations: *ALT* Alanine aminotransferase, *AST* Aspartate aminotransferase, *GGT* γ-glutamyltransferase

## Discussion

In this single-arm phase 1b study, which to our knowledge is the largest prospective trial of a checkpoint inhibitor in mACC, avelumab showed antitumor activity with an acceptable safety profile in a platinum-treated population. Three patients (6.0%) had an objective response, including patients with PD-L1+ and PD-L1− tumors, and all of whom had received only 1 (*n* = 2) or 2 (*n* = 1) prior lines of treatment. This suggests that the activity of avelumab might be greatest in patients with limited pretreatment, although the small patient numbers in this study prevent any definitive conclusion. Reasons for improved response in patients with less pretreatment may include a smaller tumor burden, decreased proportion of treatment-resistant cells within the tumor, and reduced immunosuppression associated with multiple prior lines of chemotherapy. Although the ORR and median PFS were modest in this heavily pretreated population, the disease control rate was 48.0%, median OS was 10.6 months, and the 1-year OS rate was 43.0%. No association was seen between concomitant mitotane treatment and clinical activity of avelumab, although the absence of detailed patient data relating to ongoing mitotane treatment, including drug levels, is a limitation of the study.

Apart from the current study, other data reported in ACC with anti–PD-L1/PD-1 agents are preliminary findings from phase 2 studies of nivolumab and pembrolizumab in patients with previously treated advanced ACC. Of 7 patients who received nivolumab, 5 had a best response of disease progression and 2 were awaiting evaluation [22]. Of 11 patients who received pembrolizumab, 2 had a PR, 1 achieved stable disease, and the 6-month PFS rate was 27% [23]. In addition, in the phase 1a study of avelumab in patients with various advanced malignancies, a PR occurred in a patient with ACC [13].

The manageable safety profile of avelumab seen in patients with mACC was consistent with experience in other tumor types [16–18]. Patients receiving concomitant mitotane had a higher rate of grade 3 TRAEs than those not receiving mitotane (24.0% vs 8.0%), particularly liver enzyme elevations. This reflects the known toxicity profile of mitotane, which includes hepatic, gastrointestinal, neurological, and hematologic AEs [24]. However, our study showed that the tolerability of avelumab and mitotane in combination is acceptable.

Current treatment options for patients with mACC are highly limited. In the first-line setting, response rates with mitotane monotherapy are estimated to be approximately 10% to 30%, although data from prospective trials are lacking [7]. In a randomized phase 3 study of mitotane combined with either etoposide, doxorubicin, and cisplatin or streptozocin in patients with unresectable ACC without prior treatment (except mitotane), the ORR was 23.2% vs 9.2% (*P* < .001), the disease-control rate was 58.3% vs 31.4% (*P* < .001), median PFS was 5.0 vs 2.1 months (*P* < .001), median OS was 14.8 vs 12.0 months (*P* = .07), and serious AEs occurred in 58.1% vs 41.6% of patients [25]. In a phase 2 trial of gemcitabine plus metronomic fluoropyrimidine as second-/third-line treatment in patients with advanced ACC who were receiving ongoing mitotane treatment (*n* = 28), the ORR was 7.1%, disease control rate was 46.4%, median time to progression was 5.3 months, and median OS was 9.8 months; grade 3/4 AEs were leukopenia (21.4%), thrombocytopenia (3.5%), and mucositis (3.5%) [26]. Thus, the results from our study indicate that avelumab has comparable clinical activity and may be better tolerated than existing treatment options for this hard-to-treat cancer. A randomized phase 2 study in non-small-cell lung cancer has shown that combining an anti–PD-1 antibody with platinum-based chemotherapy increased the ORR and prolonged PFS vs chemotherapy alone [27]. This suggests that studies in ACC of avelumab in combination with chemotherapy or as maintenance therapy after first-line induction chemotherapy are warranted.

Targeted molecular therapies have been explored in patients with previously treated advanced ACC, but efficacy has been poor or nonexistent. In a phase 3 trial of linsitinib (insulin-like growth factor receptor 1 and insulin receptor inhibitor) vs placebo in 139 patients with previously treated ACC, of whom 55% had received prior cisplatin, ORR was 3.3% vs 0%, disease control rate was 32.2% vs 34.7%, median PFS was 44 vs 46 days (*P* = .30), and median OS was 323 vs 356 days (*P* = .77) [28]. Treatment with erlotinib (tyrosine kinase inhibitor [TKI] of epidermal growth factor receptor) plus gemcitabine in 10 patients resulted in tumor-size reduction in 1 patient, whereas 8 patients had progressive disease at first evaluation [29]. In other studies, no patient treated with combinations of bevacizumab (anti-VEGF antibody) plus capecitabine [30] or sorafenib (multi-TKI) plus paclitaxel [31] had an objective response or stable disease. In a study of axitinib (VEGF receptor inhibitor), none of 13 patients had an objective response, 4 patients showed decreased tumor growth, and median PFS was 5.5 months [32]. Among 35 evaluable patients who received sunitinib (multi-TKI), 14.2% had stable disease as best response and median PFS was 2.8 months [33]. In a study assessing cixutumumab (anti-insulin growth factor 1 receptor antibody) plus temsirolimus (mTOR inhibitor), 11 of 26 patients (42.3%) had a best response of stable disease [34]. Thus, the activity of immunotherapy may be more promising than seen to date in studies of targeted therapies.

## Conclusions

In patients with mACC previously treated with chemotherapy, avelumab had moderate clinical activity either as monotherapy or with concurrent mitotane (50% of patients), particularly in those with limited pretreatment or PD-L1+ tumors, and with an acceptable safety profile. These findings provide a rationale for studies of avelumab-based combination treatment in patients with advanced ACC.

## Additional file

Additional file 1: **Table S1.** Confirmed best objective response based on modified immune-related response criteria. **Table S2.** Antitumor activity based on PD-L1 expression on tumor cells (1% and 5% cutoffs) in evaluable patients (*n* = 42). **Figure S1.** Computed tomography scans of a patient with adrenocortical carcinoma who experienced a long-term tumor response with avelumab treatment. The patient had a partial response documented at the second assessment (week 13) and remained on treatment without progression until last follow-up. **Figure S2.** (A) Progression-free survival (PFS) and (B) overall survival (OS) based on programmed death ligand 1 (PD-L1) expression on tumor cells (1% cutoff) in evaluable patients (*n* = 42). ACC, adrenocortical carcinoma; NE, nonevaluable. (DOCX 498 kb)

## Abbreviations

ACC: Adrenocortical carcinoma
AE: Adverse event
ALT: Alanine aminotransferase
AST: Asparate aminotransferase
CCNE1: Cyclin E1
CI: Confidence interval
CNS: Central nervous system
CR: Complete response
ECOG PS: Eastern Cooperative Oncology Group performance status
GGT: γ-glutamyltransferase
HR: Hazard ratios
mACC: Metastatic adrenocortical carcinoma
mTOR: Mammalian Target of Rapamycin
NE: Not evaluable
NF1: Neurofibromin 1
ORR: Objective response rate
OS: Overall survival
PD-1: Programmed death 1
PD-L1: Programmed death ligand 1
PFS: Progression-free survival
PRKAR1A: Protein kinase cAMP-dependent type 1 regulatory subunit alpha
RECIST: Response Evaluation Criteria In Solid Tumors
RPL22: Ribosomal protein L22
SAE: Serious adverse event
TERF2: Telomeric repeat binding factor 2
TERT: Telomerase reverse transcriptase
TKI: Tyrosine-kinase inhibitor
TRAE: Treatment-related adverse event
VEGF: Vascular endothelial growth factor
ZNRF3: Zinc and ring finger 3

## References

[1] Kebebew E, Reiff E, Duh QY, Clark OH, McMillan A (2006). Extent of disease at presentation and outcome for adrenocortical carcinoma: have we made progress?. World J Surg.

[2] Zheng S, Cherniack AD, Dewal N, Moffitt RA, Danilova L, Murray BA (2016). Comprehensive pan-genomic characterization of adrenocortical carcinoma. Cancer Cell.

[3] Colli LM, Machiela MJ, Myers TA, Jessop L, Yu K, Chanock SJ (2016). Burden of nonsynonymous mutations among TCGA cancers and candidate immune checkpoint inhibitor responses. Cancer Res.

[4] Assié G, Antoni G, Tissier F, Caillou B, Abiven G, Gicquel C (2007). Prognostic parameters of metastatic adrenocortical carcinoma. J Clin Endocrinol Metab.

[5] Fassnacht Martin, Johanssen Sarah, Quinkler Marcus, Bucsky Peter, Willenberg Holger S., Beuschlein Felix, Terzolo Massimo, Mueller Hans-Helge, Hahner Stefanie, Allolio Bruno (2008). Limited prognostic value of the 2004 International Union Against Cancer staging classification for adrenocortical carcinoma. Cancer.

[6] Lughezzani Giovanni, Sun Maxine, Perrotte Paul, Jeldres Claudio, Alasker Ahmed, Isbarn Hendrik, Budäus Lars, Shariat Shahrokh F., Guazzoni Giorgio, Montorsi Francesco, Karakiewicz Pierre I. (2010). The European Network for the Study of Adrenal Tumors staging system is prognostically superior to the international union against cancer-staging system: A North American validation. European Journal of Cancer.

[7] NCCN Clinical Practice Guidelines in Oncology. Neuroendocrine Tumors. V3. 2017. https://www.nccn.org/professionals/physician_gls/pdf/neuroendocrine.pdf. Accessed 15 Mar 2018.

[8] Libé R (2015). Adrenocortical carcinoma (ACC): diagnosis, prognosis, and treatment. Front Cell Dev Biol.

[9] Dong H, Strome SE, Salomao DR, Tamura H, Hirano F, Flies DB (2002). Tumor-associated B7-H1 promotes T-cell apoptosis: a potential mechanism of immune evasion. Nat Med.

[10] Topalian SL, Drake CG, Pardoll DM (2012). Targeting the PD-1/B7-H1(PD-L1) pathway to activate anti-tumor immunity. Curr Opin Immunol.

[11] Sunshine J, Taube JM (2015). PD-1/PD-L1 inhibitors. Curr Opin Pharmacol.

[12] Fay AP, Signoretti S, Callea M, Telό GH, McKay RR, Song J (2015). Programmed death ligand-1 expression in adrenocortical carcinoma: an exploratory biomarker study. J Immunother Cancer.

[13] Heery Christopher R, O'Sullivan-Coyne Geraldine, Madan Ravi A, Cordes Lisa, Rajan Arun, Rauckhorst Myrna, Lamping Elizabeth, Oyelakin Israel, Marté Jennifer L, Lepone Lauren M, Donahue Renee N, Grenga Italia, Cuillerot Jean-Marie, Neuteboom Berend, Heydebreck Anja von, Chin Kevin, Schlom Jeffrey, Gulley James L (2017). Avelumab for metastatic or locally advanced previously treated solid tumours (JAVELIN Solid Tumor): a phase 1a, multicohort, dose-escalation trial. The Lancet Oncology.

[14] Boyerinas B, Jochems C, Fantini M, Heery CR, Gulley JL, Tsang KY (2015). Antibody-dependent cellular cytotoxicity activity of a novel anti-PD-L1 antibody avelumab (MSB0010718C) on human tumor cells. Cancer Immunol Res.

[15] Khanna S, Thomas A, Abate-Daga D, Zhang J, Morrow B, Steinberg SM (2016). Malignant mesothelioma effusions are infiltrated by CD3^+^ T cells highly expressing PD-L1 and the PD-L1^+^ tumor cells within these effusions are susceptible to ADCC by the anti-PD-L1 antibody avelumab. J Thorac Oncol.

[16] Kaufman HL, Russell J, Hamid O, Bhatia S, Terheyden P, D'Angelo SP (2016). Avelumab in patients with chemotherapy-refractory metastatic Merkel cell carcinoma: a multicentre, single-group, open-label, phase 2 trial. Lancet Oncol.

[17] Gulley James L, Rajan Arun, Spigel David R, Iannotti Nicholas, Chandler Jason, Wong Deborah J L, Leach Joseph, Edenfield W Jeff, Wang Ding, Grote Hans Juergen, Heydebreck Anja von, Chin Kevin, Cuillerot Jean-Marie, Kelly Karen (2017). Avelumab for patients with previously treated metastatic or recurrent non-small-cell lung cancer (JAVELIN Solid Tumor): dose-expansion cohort of a multicentre, open-label, phase 1b trial. The Lancet Oncology.

[18] Apolo AB, Infante JR, Balmanoukian A, Patel MR, Wang D, Kelly K (2017). Avelumab, an anti-programmed death-ligand 1 antibody, in patients with refractory metastatic urothelial carcinoma: results from a multicenter, phase Ib study. J Clin Oncol.

[19] Bavencio (avelumab) [package insert]. Rockland, MD: EMD Serono, Inc; 2017.

[20] Eisenhauer E.A., Therasse P., Bogaerts J., Schwartz L.H., Sargent D., Ford R., Dancey J., Arbuck S., Gwyther S., Mooney M., Rubinstein L., Shankar L., Dodd L., Kaplan R., Lacombe D., Verweij J. (2009). New response evaluation criteria in solid tumours: Revised RECIST guideline (version 1.1). European Journal of Cancer.

[21] Wolchok JD, Hoos A, O’Day S, Weber JS, Hamid O, Lebbé C (2009). Guidelines for the evaluation of immune therapy activity in solid tumors: immune-related response criteria. Clin Cancer Res.

[22] Cavalcante L, Carneiro BA, Costa RLB, Chae YK, Rademaker A, Giles FJ (2017). Preliminary results from a phase II study of nivolumab for patients with metastatic adrenocortical carcinoma (ACC). J Clin Oncol.

[23] Habra MA, Campbell M, Jimenez C, Karp D, Hong D, Subbiah V (2017). Efficacy of pembrolizumab (MK-3475) in patients with adrenocortical carcinoma. J Immunother Cancer.

[24] Tacon LJ, Prichard RS, Soon PS, Robinson BG, Clifton-Bligh RJ, Sidhu SB (2011). Current and emerging therapies for advanced adrenocortical carcinoma. Oncologist.

[25] Fassnacht M, Terzolo M, Allolio B, Baudin E, Haak H, Berruti A (2012). Combination chemotherapy in advanced adrenocortical carcinoma. N Engl J Med.

[26] Sperone P, Ferrero A, Daffara F, Priola A, Zaggia B, Volante M (2010). Gemcitabine plus metronomic 5-fluorouracil or capecitabine as a second−/third-line chemotherapy in advanced adrenocortical carcinoma: a multicenter phase II study. Endocr Relat Cancer.

[27] Langer CJ, Gadgeel SM, Borghaei H, Papadimitrakopoulou VA, Patnaik A, Powell SF (2016). KEYNOTE-021 investigators. Carboplatin and pemetrexed with or without pembrolizumab for advanced, non-squamous non-small-cell lung cancer: a randomised, phase 2 cohort of the open-label KEYNOTE-021 study. Lancet Oncol.

[28] Fassnacht M, Berruti A, Baudin E, Demeure MJ, Gilbert J, Haak H (2015). Linsitinib (OSI-906) versus placebo for patients with locally advanced or metastatic adrenocortical carcinoma: a double-blind, randomised, phase 3 study. Lancet Oncol.

[29] Quinkler M, Hahner S, Wortmann S, Johanssen S, Adam P, Ritter C (2008). Treatment of advanced adrenocortical carcinoma with erlotinib plus gemcitabine. J Clin Endocrinol Metab.

[30] Wortmann S, Quinkler M, Ritter C, Kroiss M, Johanssen S, Hahner S (2010). Bevacizumab plus capecitabine as a salvage therapy in advanced adrenocortical carcinoma. Eur J Endocrinol.

[31] Berruti A, Sperone P, Ferrero A, Germano A, Ardito A, Priola AM (2012). Phase II study of weekly paclitaxel and sorafenib as second/third-line therapy in patients with adrenocortical carcinoma. Eur J Endocrinol.

[32] O’Sullivan C, Edgerly M, Velarde M, Wilkerson J, Venkatesan AM, Pittaluga S (2014). The VEGF inhibitor axitinib has limited effectiveness as a therapy for adrenocortical cancer. J Clin Endocrinol Metab.

[33] Kroiss M, Quinkler M, Johanssen S, van Erp NP, Lankheet N, Pöllinger A (2012). Sunitinib in refractory adrenocortical carcinoma: a phase II, single-arm, open-label trial. J Clin Endocrinol Metab.

[34] Naing A, Lorusso P, Fu S, Hong D, Chen HX, Doyle LA (2013). Insulin growth factor receptor (IGF-1R) antibody cixutumumab combined with the mTOR inhibitor temsirolimus in patients with metastatic adrenocortical carcinoma. Br J Cancer.

