# Supplementary material for: Avelumab in patients with previously treated metastatic adrenocortical carcinoma: phase 1b results from the JAVELIN solid tumor trial
Source: J Immunother Cancer. 2018 Oct 22;6:111. doi: 10.1186/s40425-018-0424-9 (PMC6198369; doi:10.1186/s40425-018-0424-9)

**SUPPLEMENTARY MATERIAL**

**Supplementary Table S1.** Confirmed Best Objective Response Based on Modified Immune-Related Response Criteria

| **Response** | **(N=50)** |
| --- | --- |
| Confirmed best overall response, n (%) |  |
| Immune-related complete response | 0 |
| Immune-related partial response | 3 (6.0) |
| Immune-related stable disease | 26 (52.0) |
| Immune-related progressive disease | 14 (28.0) |
| Nonevaluable | 7 (14.0) |
| Immune-related ORR, % (95% CI) | 6.0 (1.3–16.5) |

Abbreviation: ORR, objective response rate.

**Supplementary Table S2.** Antitumor Activity Based on PD-L1 Expression on Tumor Cells (1% and 5% cutoffs) in Evaluable Patients (n=42)

| **PD-L1 Cutoff** | **PD-L1+ Tumor** | **PD-L1− Tumor** | ***P* Value** | **Hazard Ratio (95% CI)** |
| --- | --- | --- | --- | --- |
| **≥1% tumor cells** | | | | |
| Patients, n | 15 | 27 | — | — |
| ORR (95% CI), % | 13.3  (1.7–40.5) | 3.7  (0.1–19.0) | .287 | — |
| Median PFS (95% CI), months | 2.1  (1.3–6.8) | 1.8  (1.4–4.0) | — | 0.89  (0.45–1.76) |
| Median OS (95% CI), months | 9.1  (7.3–14.4) | 11.5  (8.0–NE) | — | 1.30  (0.52–3.24) |
| **≥5% tumor cells** | | | | |
| Patients, n | 12 | 30 | — | — |
| ORR (95% CI), % | 16.7  (2.1–48.4) | 3.3  (0.1–17.2) | .192 | — |
| Median PFS (95% CI), months | 5.5  (1.3–8.2) | 1.7  (1.4–4.0) | — | 0.66  (0.32–1.39) |
| Median OS (95% CI), months | 14.4  (7.4–14.4) | 10.6  (7.3–NE) | — | 0.91  (0.33–2.49) |

Abbreviations: NE, not estimable; ORR, objective response rate; OS, overall survival; PD-L1, programmed death ligand 1; PFS, progression-free survival.

**Supplementary Figure S1.** Computed tomography scans of a patient with adrenocortical carcinoma who experienced a long-term tumor response with avelumab treatment. The patient had a partial response documented at the second assessment (week 13) and remained on treatment without progression until last follow-up.


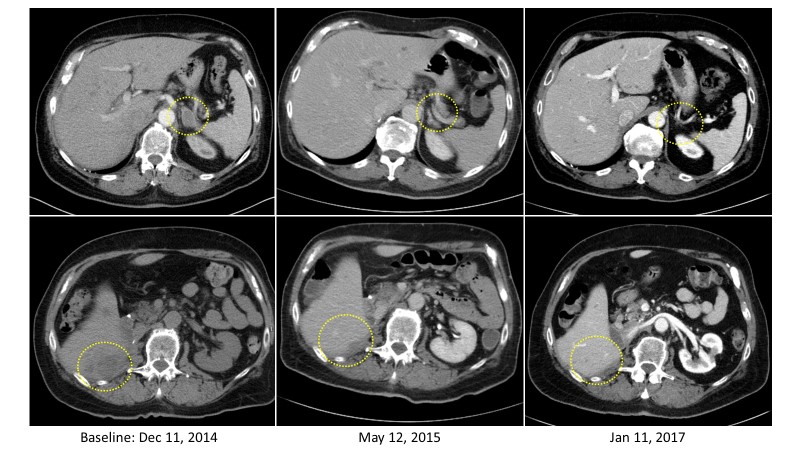


**Supplementary Figure S2.** (A) Progression-free survival (PFS) and (B) overall survival (OS) based on programmed death ligand 1 (PD-L1) expression on tumor cells (1% cutoff) in evaluable patients (n=42). ACC, adrenocortical carcinoma; NE, nonevaluable.

**A**


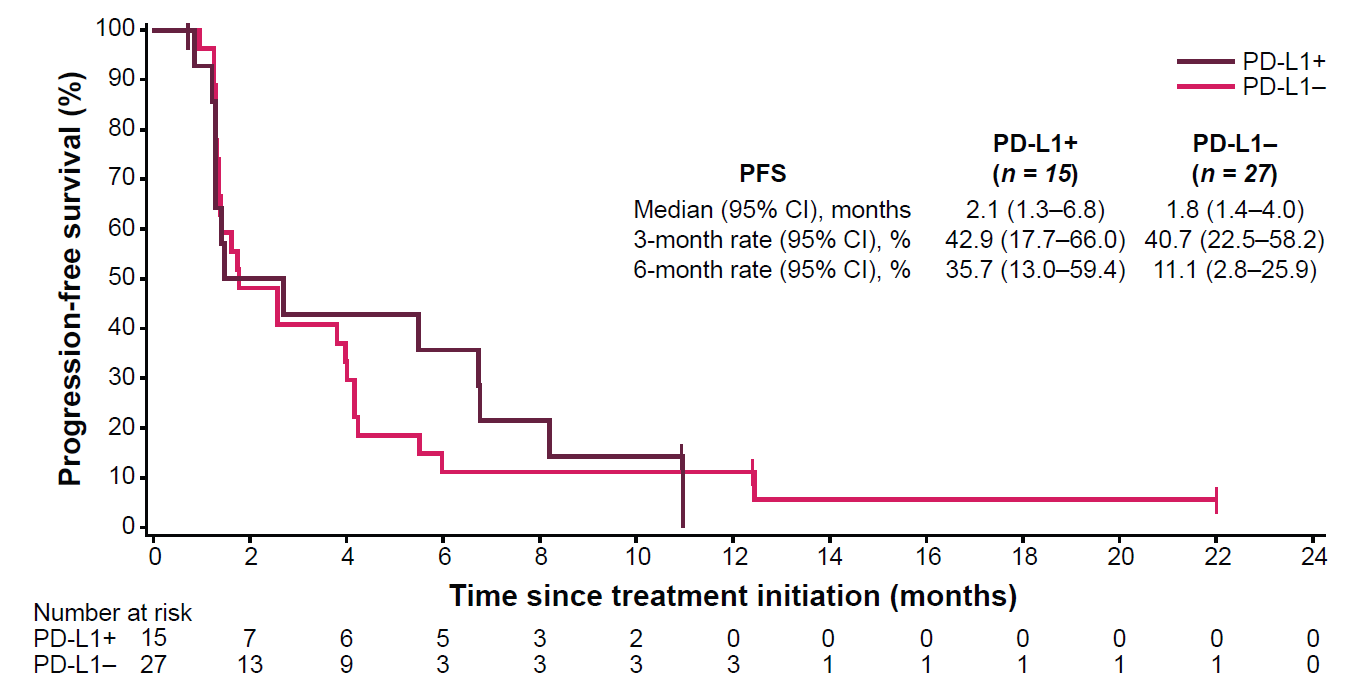
**B**
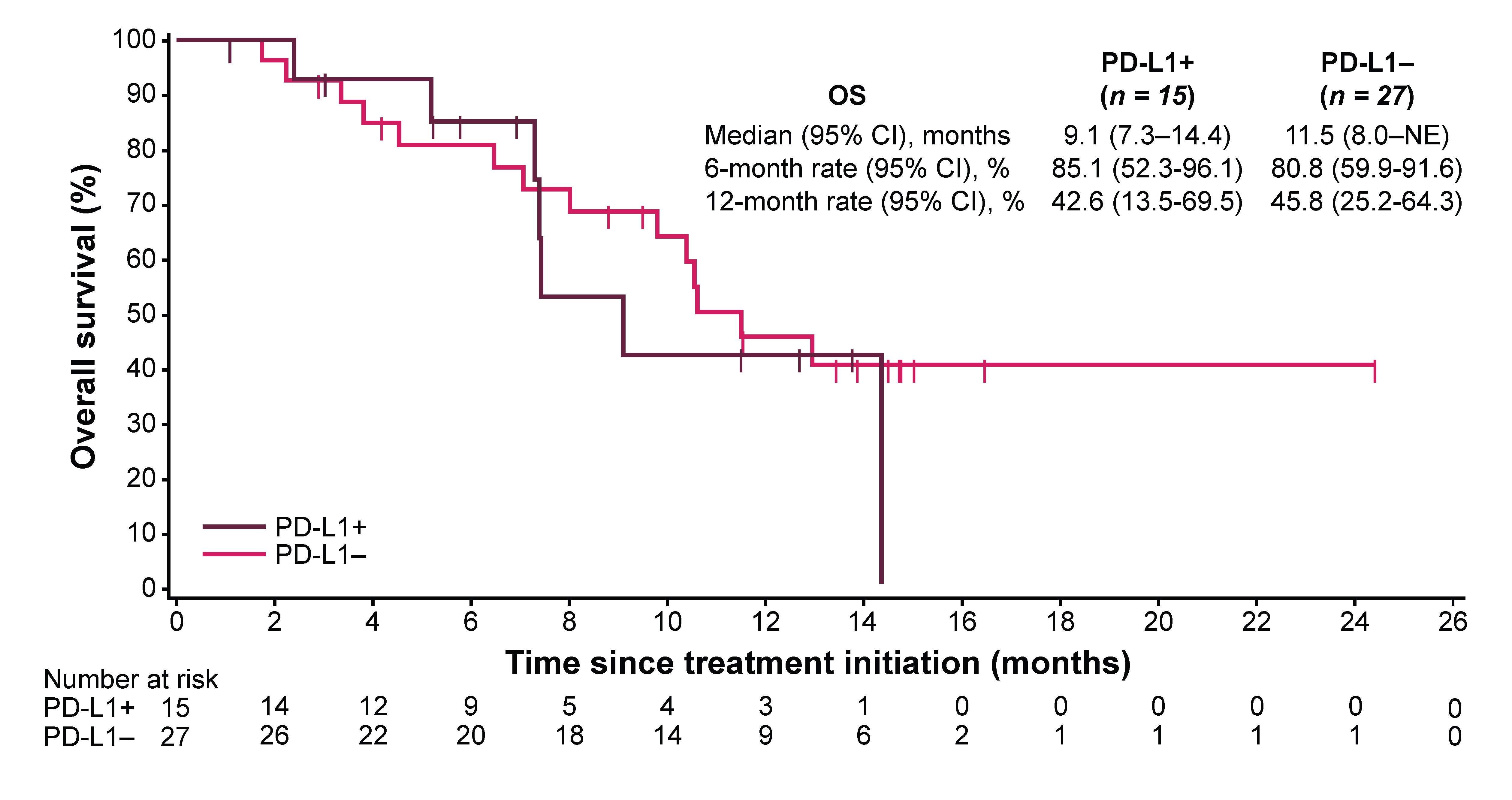

Supplement: Supplementary file 1 — Table S1. Confirmed best objective response based on modified immune-related response criteria. Table S2. Antitumor activity based on PD-L1 expression on tumor cells (1% and 5% cutoffs) in evaluable patients (n = 42). Figure S1. Computed tomography scans of a patient with adrenocortical carcinoma who experienced a long-term tumor response with avelumab treatment. The patient had a partial response documented at the second assessment (week 13) and remained on treatment without progression until last follow-up. Figure S2. (A) Progression-free survival (PFS) and (B) overall survival (OS) based on programmed death ligand 1 (PD-L1) expression on tumor cells (1% cutoff) in evaluable patients (n = 42). ACC, adrenocortical carcinoma; NE, nonevaluable. (DOCX 498 kb) [file 40425_2018_424_MOESM1_ESM.docx]
